# Supplementary figures and images for: Modeling and predicting the overlap of B- and T-cell receptor repertoires in healthy and SARS-CoV-2 infected individuals
Source: PLoS Genet. 2023 Feb 24;19(2):e1010652. doi: 10.1371/journal.pgen.1010652 (PMC10075420; doi:10.1371/journal.pgen.1010652)

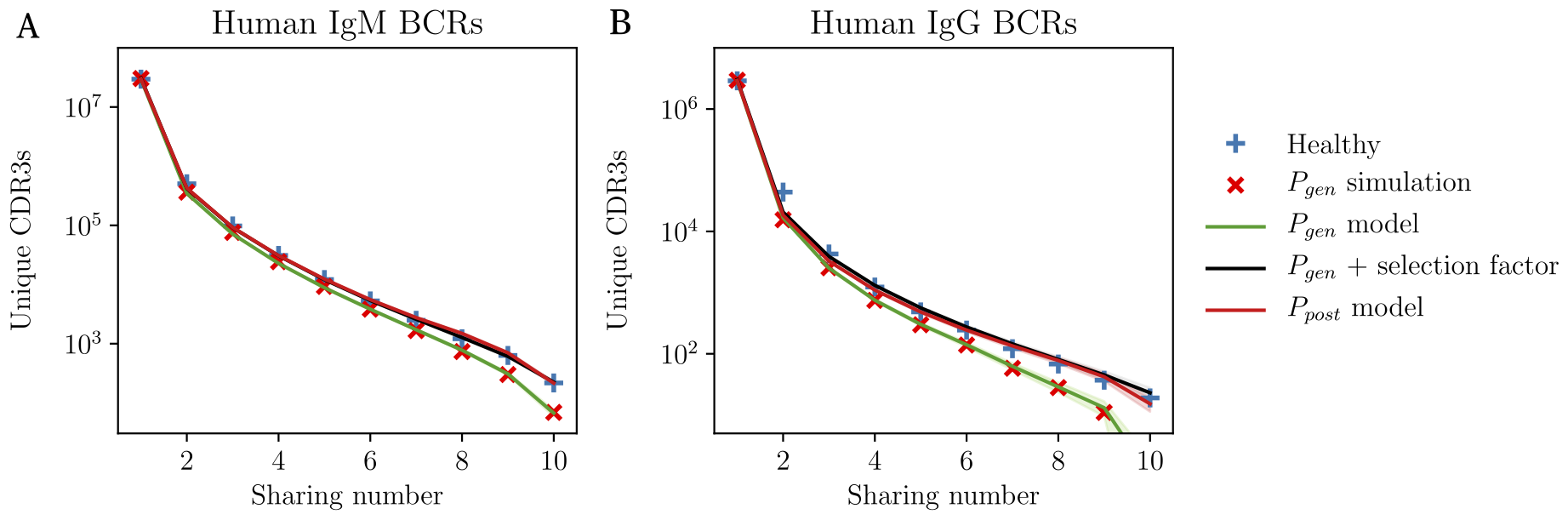

Supplement: S1 Fig — (A) Distribution of the sharing number (the number of individuals in which a sequence is seen) of CDR3 amino-acid sequences of the heavy chains of IgM repertoires from 10 individuals. The prediction from the raw recombination model (green line corresponds to model predictions using the Pgen distribution while red crosses are drawn from sequence simulation using thePgen model) underestimates sharing. Adding an ad hoc correction factor assuming a fraction q of sequences passing selection (q = 0.759 ± 0.001) gives a good fit to the data (black curve). The prediction from the generation and selection models (Ppost, red line) reproduces the curve perfectly, with no need for a correction factor. (B) Distribution of sharing number for the IgG repertoires of the same donors. The analysis is done on the naive ancestors of reconstructed clonal lineages. The Pgen model is again inaccurate, requiring a correction factor q = 0.636 ± 0.005, while Ppost works well. As one can observe, the selection factor decreases its value from IgM to IgG and since the smaller the q value the stronger the selection, this parameter turns out to be quantifying the selection that accompanies class switching. (TIFF) [file pgen.1010652.s001.tiff]

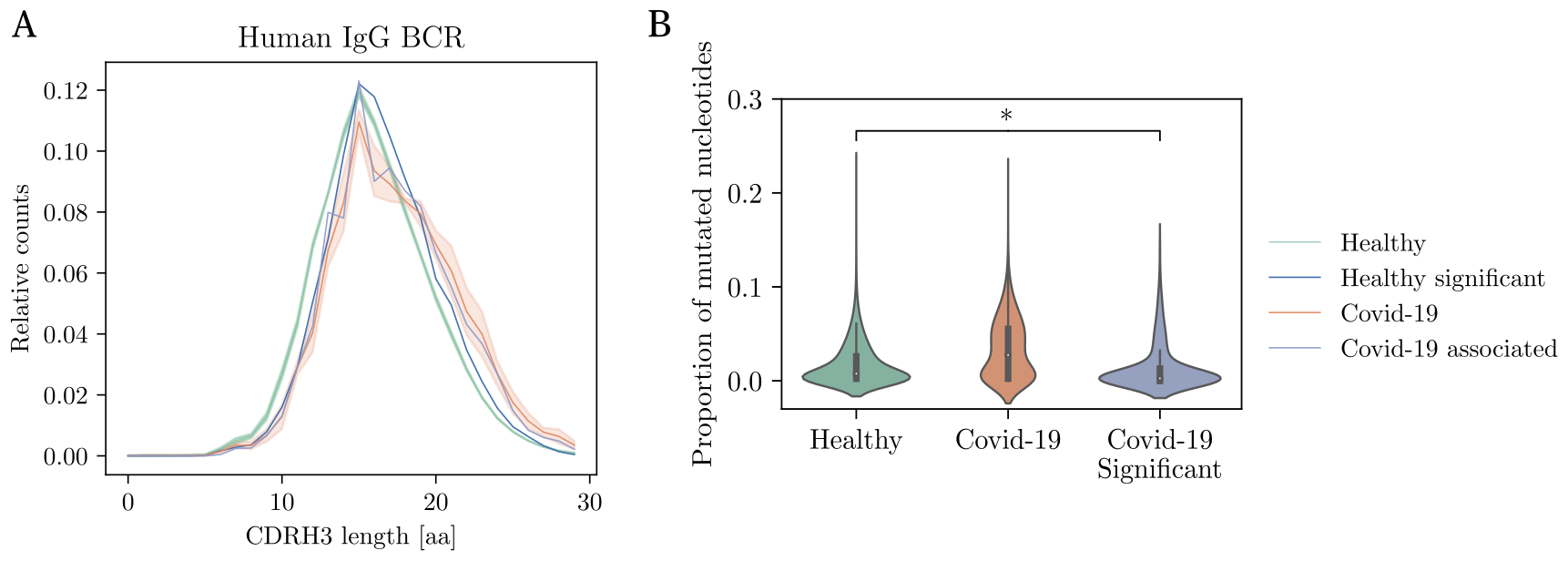

Supplement: S2 Fig — (A) Distribution of the sharing numberof CDR3 + V gene + J gene amino-acid sequences of the heavy chains of IgM repertoires from 10 individuals. Even though using this more stringent definition of sharing the number of public clonotypes remarkably decreases, the prediction from the generation and selection models (Ppost, red line) still reproduces the curve perfectly. (B) Distribution of sharing number (CDR3 + V gene + J gene) for the IgG repertoires of the same healthy donors and model predictions from Ppost model simulations. (C) Distribution of sharing number (CDR3 + V gene + J gene) for the IgG repertoires of the SARS-CoV-2 infected donors and model predictions from Ppost model simulations. (TIFF) [file pgen.1010652.s002.tiff]

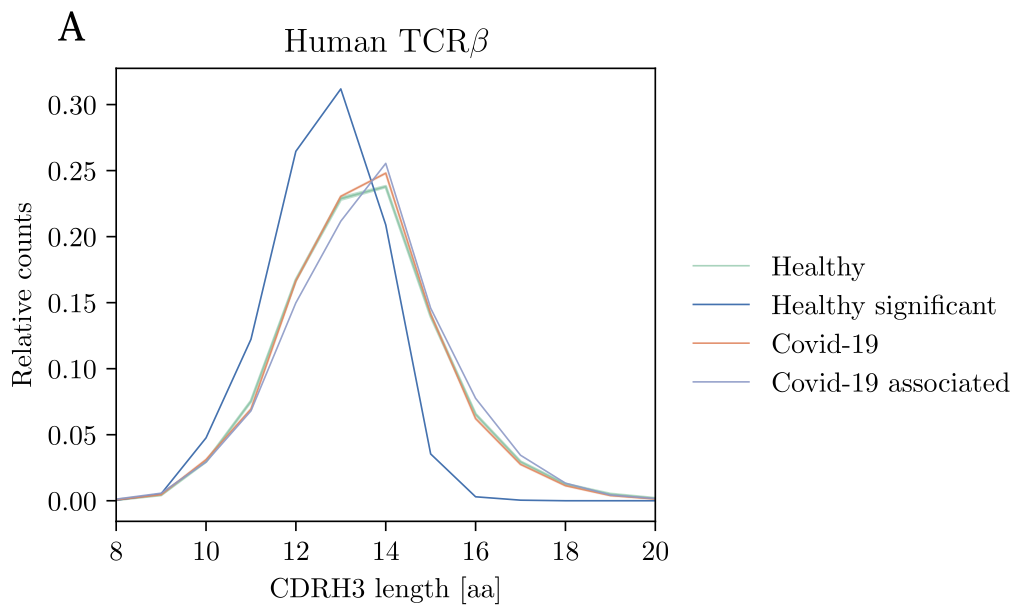

Supplement: S3 Fig — (A) CDRH3 length distribution averaged over individuals within each cohort of B cell receptors. A control was added for sequences found to be significantly more shared than expected in healthy individuals (named as healthy significant, blue curve), to account for the bias that shared sequences tend to have a shorter CDR3. (B) Violin plots representing the proportion of mutated nucleotides in B cell receptor repertoires per individual in healthy and COVID-19 cohorts, as well as in significantly shared sequences. The dot represents the median SHM percentage. Student’s t test: *p < 0.05. (TIFF) [file pgen.1010652.s003.tiff]

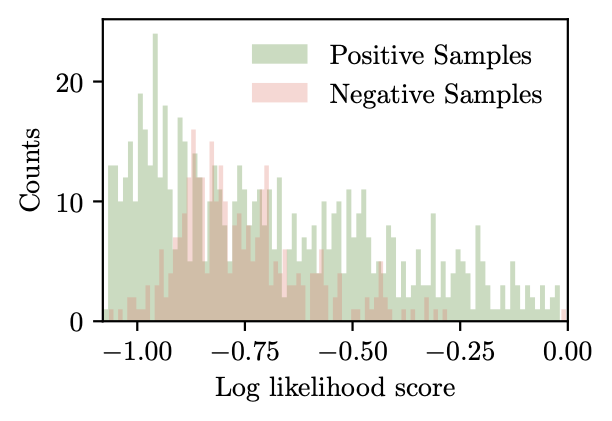

Supplement: S4 Fig — (A) CDRH3 length distributions of generic and significantly overshared TCR sequences. Significantly overshared sequences in healthy individuals (healthy significant, blue curve) are much shorter than significantly overshared sequences in COVID-19 individuals (COVID-19 associated, purple curve). (TIFF) [file pgen.1010652.s004.tiff]

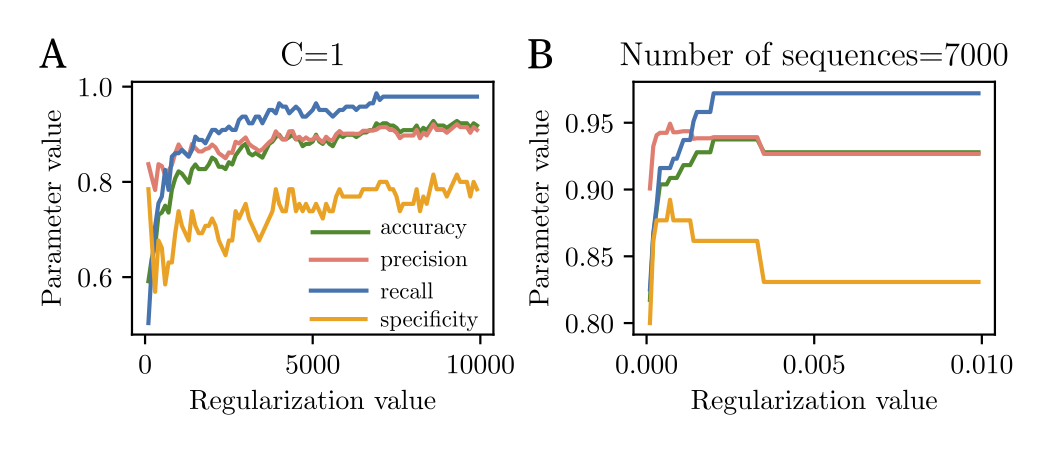

Supplement: S5 Fig — Distribution of log-likelihood ratio scores for 1000 T-cell repertoires belonging to the test cohort of 700 COVID-19 patients and 300 healthy individuals. The distribution of scores for each group completely overlaps, leading to a classifier with a very poor accuracy. (TIFF) [file pgen.1010652.s005.tiff]

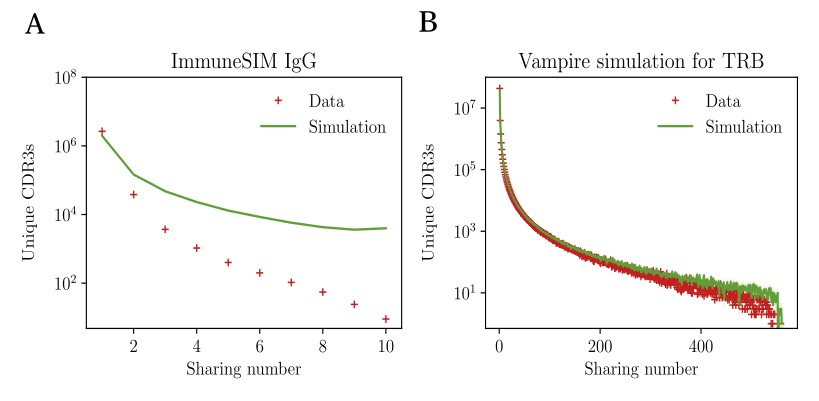

Supplement: S6 Fig — (A) Variation of the logistic regression performance as a function of the total number of sequences. In the absence of regularization, both precision and accuracy reach their maximum for n = 7000 sequences. (B) For the optimal number of sequences found, the precision and specificity are maximized for C = 0.0008. (TIFF) [file pgen.1010652.s006.tiff]

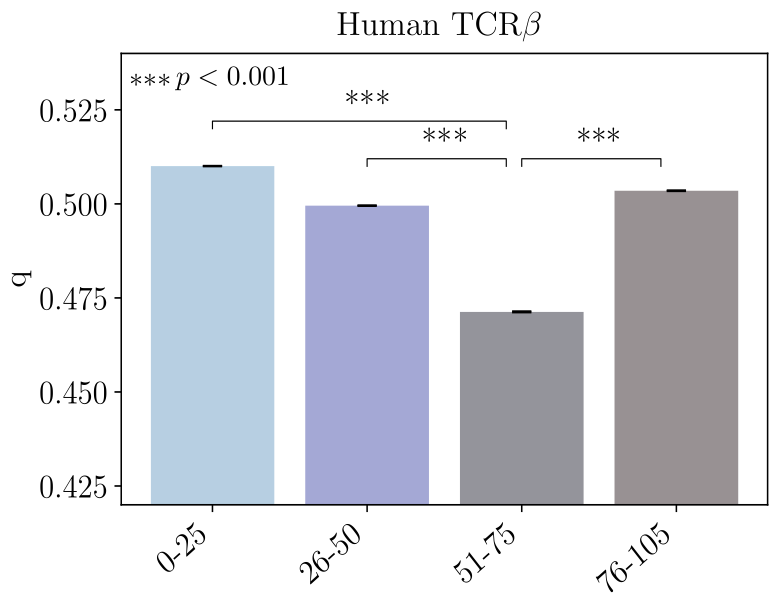

Supplement: S7 Fig — (A) Distribution of the sharing number of CDR3 amino-acid sequences of IgG repertoires from 10 individuals. The prediction from the model (green line) comes from sequences simulated using the immuneSIM software [21]. The simulation dramatically diverges from the real amount of shared sequences indicating either a bad recombination model or an overestimation of selection. (B) Distribution of the sharing number of CDR3 amino-acid sequences of TCRβ from 666 patients. Model prediction (green line) was obtained using the default model of vampire [23] trained on the same data set. vampire slightly overestimates the sharing distribution. (TIFF) [file pgen.1010652.s007.tiff]

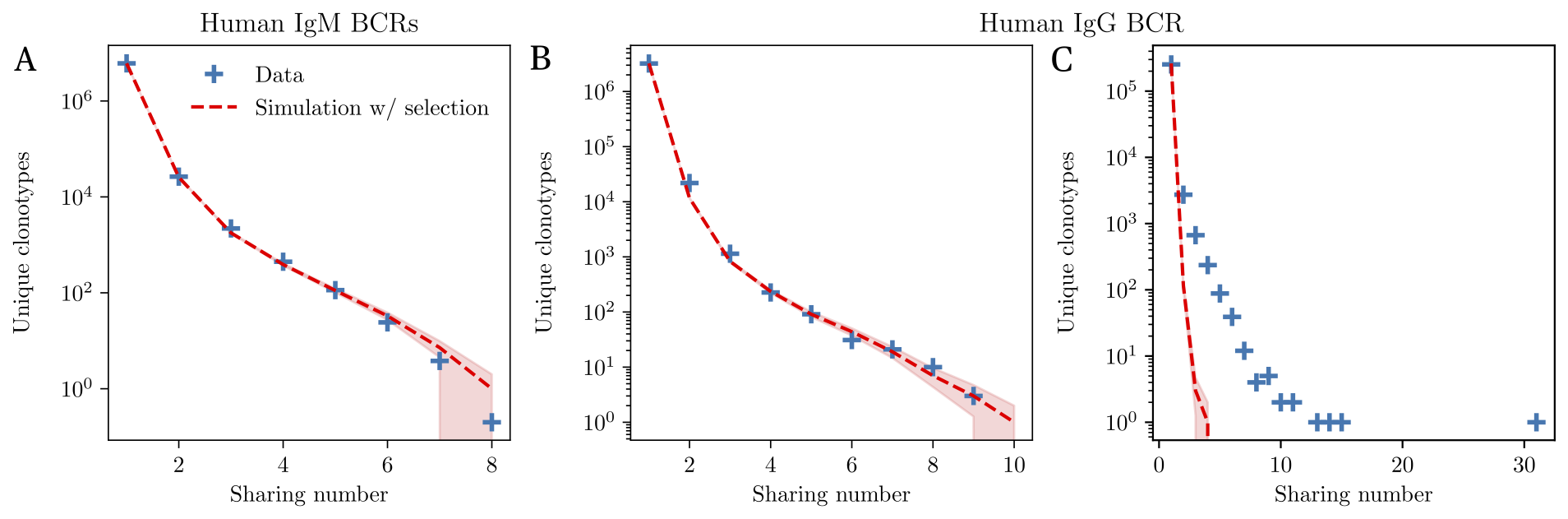

Supplement: S8 Fig — Values of the selection parameter q as a function of age in repertoires of an independent cohort from [31]. Again, diversity significantly (p<0.001) decreases in repertoires of older people, supporting the idea that aging is accompanied by a loss of repertoire diversity, with the exception of individuals of age 75+. (TIFF) [file pgen.1010652.s008.tiff]
