# Supplementary material for: Modeling and predicting the overlap of B- and T-cell receptor repertoires in healthy and SARS-CoV-2 infected individuals
Source: PLoS Genet. 2023 Feb 24;19(2):e1010652. doi: 10.1371/journal.pgen.1010652 (PMC10075420; doi:10.1371/journal.pgen.1010652)
Supplement: Attachment — Submitted filename: PLoS Genetics sharing paper response.pdf [file pgen.1010652.s013.pdf]

We thank the reviewers and the editor for constructive feedback. We have addressed all the reviewer comments and added new analysis following their suggestions. In this response, we quote the reviewer report in italic. Our response to the comments is in standard font. We have uploaded a revised manuscript file with all changes highlighted in red, along with an all black new version of the manuscript.

Comments to the Authors:

Reviewer #1: In this work, Ortega and colleagues analyse overlap of TCR/BCR repertoires among S-CoV-2 neg and pos individuals using statistical models.

I don't have many comments. The main comment is: how does this work conceptually differ from previous works of the research group? The research group has recently published on both Covid repertoires as well statistical models for generation and sharing of immune receptors. What exactly is the novelty in this work as compared to the group's previous research as well as to other works in this space. Can this be made more clear?

For example, this sentence from the abstract "Yet many of these public receptors are shared by chance. We present a statistical approach, defined in terms of a probabilistic V(D)J recombination model enhanced by a selection factor, that describes repertoire diversity and predicts with high accuracy the spectrum of repertoire overlap in healthy individuals. " sounds very similar to previous manuscripts from this group. Please reformat the manuscript to isolate the advance made in this manuscript. Or, if there is no conceptual advance made here, rewrite to focus on the biological insight gained by the data analysis (but also there explain how it is novel compared to prior work in this space).

We thank the Reviewer for their positive assessment of our work and for pointing out possible confusion with previous work. There are many differences between the current manuscript and previous work from our group.

First, here we look at sharing in functional repertoires, and our theoretical prediction includes sequence-specific selection models on top of the probability of TCR/BCR generation. These selection models account for receptor functionality checks and central tolerance, and can pick up amino acid specific biases. Accounting for this sequence specific factor is necessary for predicting the sharing distributions, and we have never included it before. Before (Elhanati 2018) we used a simplified global bias factor that we show in FIg S1 – it does ok but not as well for TCR but badly for BCR.

Second, while our previous work on sharing was restricted to TCR, here we generalize it to BCR, dealing with the specific challenge of considering the sharing of ancestral (unmutated) sequences.

Third, here we study the oversharing of receptors in COVID patients, and not just healthy patients as in our previous sharing study. While we have published work regarding COVID dynamical response in TCR, it did not have any sharing analysis, as it was focused on just 2 donors.

Lastly, we also perform sequence and network analysis, identifying features and logos linked to response, which we cross-validate with known B/TCRs from databases. Based on that, we also propose a score for COVID19 positivity based on the repertoire only.

We have rewritten the abstract and the introduction to make these differences more clear.

Minor:

Figure 1 looks a bit disorganized and could benefit from streamlining.

We have reorganized Figure 1.

Reviewer #2*: In their manuscript "Modeling and predicting the overlap of B- and T-cell receptor repertoires in healthy and SARS-CoV-2 infected individuals" Dr. Ruiz Ortega et al. describes bioinformatic processes to investigate public B and T cell repertoires, an approach that was also applied to studies of immune repertoires identified in subjects infected with SARS-CoV-2. They provide a convincing analysis framework, but one that also, as other pipelines designed to interrogate big data, requires further assessment.*

We thank the Reviewer for their positive assessment of our work.

1. In several instances the focus of the study is entirely on CDR3. This is a common approach in particular in studies of T cell repertoires. CDR3 is certainly at the centre of determination of specificity but it operates in the context of other sequences, such as those encoded by the IGHV. The light chain is of course also a key feature of factors that determine specificity but, as the authors rightfully describe, it is currently not economically feasible to collect sufficient single cell data to carry out a study like this. However, the authors ought to take in particular sequences encoded by IGHV into account for instance in studies of SARS-CoV-2-specific antibodies.

We have redone the BCR analysis in Fig 2 (healthy) and Fig 3 (SARS-CoV-2) by including IGHV and IGHJ gene usage on top of the CDR3. The results are shown in Supplementary Figures S2. As expected, we see a notable drop in the number of shared clonotypes compared to the analysis with CDR3 only. Nevertheless, the sharing distribution remains completely predicted by our model for healthy patients, while it strongly underestimates sharing in COVID19 patients. We also extended our analysis of identifying SARS-CoV-2 specific clonotypes to include V and J genes, and validated them against the literature-based database, again observing a significantly larger overlap than with random receptor sequences. Therefore, for all of the above analysis, our conclusions do not change. We have added text describing the new results in the main text, on page 4 and page 7.

2. In some instances, the authors do to take sequences encoded by the V gene into consideration. Annotation of such data is complicated by the fact that the TRBV and IGHV loci are highly variable in terms of structural variation, gene duplication and the existence of highly similar alleles in different gene locations. How does the analytical approach deal with these aspects, for instance but not restricted to genes like IGHV3-30/3-30-3/3-30-5/3-33?

We thank the Reviewer for pointing out this point to us, which allows us to clarify it. Whenever comparing V or J genes, we use a strict definition of identity of the germline gene, and not

similarity. In our new analysis of full amino-acid sequence sharing, we make clear that we define sharing in that case by imposing exact identity of the V and J gene assignment (bottom of page 4).

3. The authors identify a set of non-productive sequences that are used for some comparisons. These reads might have an origin in non-productive transcripts but may also be a consequence of PCR or sequencing errors. How does the authors deal with these matters to ensure that the reads are truly non-functional in the B cell population and not technical artefacts?

Indels as a consequence of PCR or sequencing errors are extremely rare, as has been previously assessed on the V gene portion of TCR. For BCR, one might be worried about hypermutation indels. We have previously estimated the per-bp rate of insertions and deletions in the VH segments of productive IgG to be ~1e-4 (Lupo et al, PLoS CB 2022). Based on this rate we estimate the expected rate of indels in CDR3 to be around 50bp x 1e-4 ~ 0.5%, which is much smaller than the number of frame-shifted CDR3. We conclude that the probability of an indel (either appearing as a biological mutation or from PCR) is very small and it can be neglected. We now include these estimates and this discussion in the Methods.

4. Public clonotypes seems often to be associated with IGHJ6 (see for instance Figure 3D, Figure 4 and Suppl Fig 2A). This might be a technical artifact. The alleles of IGHJ6 adds a particularly long stretch of residues into CDR3, thereby allowing the for a higher similarity score in the bioinformatic pipeline that allows sequences to reach the identity cutoff. The authors must comment on this.

We agree that the fact that public clonotypes often have IGHJ6 may be an artefact. However, this is exactly what the comparison with the model prediction accounts for. Our analysis is based on the Pgen estimate, which accounts for the unusual long stretch into CDR3, or any other bias of the generation process. We have added a comment about this. Also, since we do not use a similarity score, there is no possibility of error coming from the cut-off.

5. In Figure 4 the authors show CDR3s that differ in length. These differences might certainly be a consequence of insertion/deletion hypermutation events but are more likely a consequence of differential V-DJ or D-J splicing events that exploit similar D/J genes. The frequency of some of the length differences suggests that they do not represent insertion/deletion events. Differences in length of for instance clones 1, 5, 7, 9, 14, 16, 17, 18, 20, 21, 23, 24 are indicative of such artifacts. That puts the true clonal relationship of these groups into questions. Are they really clonally related or just similar clones that happens to represent common sequences. It is not obvious to me if they were derived each from a single subject or derived from multiple individuals; please clarify.

We thank the Reviewer for the comment. Figure 4 does not show lineages but graphs of neighboring CDR3 in aa space. The underlying idea is that CDR3 similar in aa sequence are likely to bind the same target, not that they come from the same progenitor (they might even be found in distinct donors). We have now clarified this point when defining clusters.

6. Sequences that carry long IGHJ genes but also those that incorporate long IGHD might be better at passing thresholds of the analysis pipeline as N nucleotides represents a smaller fraction of the CDR3. Do the authors identify such aspects of the analysis (for instance, are

incorporated D genes of public clones longer than those typically found in rearrangements as such a finding identifies a limitation of the pipeline)?

Since our analysis is based on Pgen and SONIA, these points are corrected for. Pgen accounts for any abnormally long D genes and SONIA takes the CDR3 into account amino acid by amino acid without taking into account the identity of the D gene. We have added a short explanation of this point.

7. In Figure 4 it is stated that light chain CDR3 conservation is even more remarkable that that of heavy chains. This is entirely expected as these CDR3 show much less diversity in general and depend only on the V-J rearrangement and a limited incorporation of N nucleotides. Human TRBV rearrangements use only a very limited set of D genes but more extensive V gene trimming (as compared to IGHV). How does this affect analysis?

Thank you for pointing this out. We have added a comment about this.

8. CDR3 regions similar to those of SARS-CoV-2 specific clones in an antibody database have been identified (page 7/32). Do these sequences also share V gene? Even if CDR3 is an important part of generation of the specificity it is commonly dependent on other features of the antibodies sequence. We cannot in this context relate to the light chain but the authors would be able to relate to the V-gene.

In response to point 1 we examined the overlap between overshared sequences, and public databases of COVID sequences, imposing VJ gene identity as well as CDR3 similarity throughout the analysis.

In a separate check, looking back at the overshared COVID-associated CDR3 and their overlap with the COVID database, we asked whether they also had the same V gene, in at least some of the donors. We found that a majority of them did, and in higher fraction than would be expected by chance. This check is also added to the main text.

9. On page 7 (line 227 the authors state (2.7±1.5 sequences). What does it represent in relative terms?

We have rewritten this statement and divided the number of overlapping sequencing by the number of sequences to obtain 0.04%.

10. In several instances including in Figure 3 and in the text the authors state that they observe a difference but that it is not statistically significant. It would be much better to say something like "the data was not sufficiently powered to with certainty identify a statistically significant difference between the groups".

We have followed the Reviewers suggestion. Thank you.

11. Are all data sets used full length or do they cover only a part of the IGHV and TRBV genes? If the latter is the case, this may affect gene assignment and clonal binning. Please comment how this may affect analysis and results.

The BCR datasets used start in the CDR1 region of the V gene, so at the very beginning of the sequences, so that alignment is never ambiguous. We have added a comment in the methods about this.

12. Public antibodies have been identified in antibodies against the S-protein / RBD of SARS-CoV-2. Many of the public clones recognize early strains of the virus but not omicron. The present data sets, were they generated from samples collected during the early phase of the pandemic or following circulating of mutated viruses? If some data sets were collected at a point in time when other strains were dominant, this may impact the outcome of analysis. Please discuss how this relates to the results.

This is all wild-type, as the studies all predate omicron. The entire analysis is for the original strain and we cannot comment on how it would be affected for omicron without new experiments. We clarified in the text that the data was collected before omicron.

13. On line 455 the authors relate to a particular, common CDR3. This sequence has been generated using very little (if any) incorporation of a D gene and limited N nucleotide addition. I can myself identify this sequence in IgM data sets in association with different IGHV genes like IGHV1-2 and IGHV3-74. It is thus not unexpected that it is identified. It may however not relate to one but several clonotypes. Please discuss or delete this particular part of the discussion.

We have added text and a reference to discuss that point. We could find 25 V genes associated with that CDR3, But in fact, the most common association (32/89) was with IGHV4-59, consistent with previous reports. We stress here again that the COVID-specific sequences are overshared relative to a baseline expectation that is well predicted by our model, and which already takes into account that some CDR3 are restricted by some common V choice.

14. The authors may consider shortening the manuscripts as it is quite extensive, e.g. but not limited to the paragraph starting on line 503.

Thank you for the suggestion. We have decided to keep it as is, for clarity and to address all the comments by the reviewers.
